# α-Glucosidase and Protein Tyrosine Phosphatase 1B Inhibitory Activity of Plastoquinones from Marine Brown Alga *Sargassum serratifolium*

**DOI:** 10.3390/md15120368

**Published:** 2017-12-01

**Authors:** Md. Yousof Ali, Da Hye Kim, Su Hui Seong, Hyeung-Rak Kim, Hyun Ah Jung, Jae Sue Choi

**Affiliations:** 1 Department of Food and Life Science, Pukyong National University, Busan 48513, Korea; yousufbge@gmail.com (M.Y.A.); md2253@naver.com (D.H.K.); seongsuhui@naver.com (S.H.S.); hrkim@pknu.ac.kr (H.-R.K.); 2 Department of Food Science and Human Nutrition, Chonbuk National University, Jeonju 54896, Korea

**Keywords:** *Sargassum serratifolium*, PTP1B, α-glucosidase, plastoquinones, molecular docking simulation

## Abstract

*Sargassum serratifolium* C. Agardh (Phaeophyceae, Fucales) is a marine brown alga that belongs to the family Sargassaceae. It is widely distributed throughout coastal areas of Korea and Japan. *S. serratifolium* has been found to contain high concentrations of plastoquinones, which have strong anti-cancer, anti-inflammatory, antioxidant, and neuroprotective activity. This study aims to investigate the anti-diabetic activity of *S. serratifolium* and its major constituents through inhibition of protein tyrosine phosphatase 1B (PTP1B), α-glucosidase, and ONOO^−^-mediated albumin nitration. *S. serratifolium* ethanolic extract and fractions exhibited broad PTP1B and α-glucosidase inhibitory activity (IC_50_, 1.83~7.04 and 3.16~24.16 µg/mL for PTP1B and α-glucosidase, respectively). In an attempt to identify bioactive compounds, three plastoquinones (sargahydroquinoic acid, sargachromenol and sargaquinoic acid) were isolated from the active *n*-hexane fraction of *S*. *serratifolium*. All three plastoquinones exhibited dose-dependent inhibitory activity against PTP1B in the IC_50_ range of 5.14–14.15 µM, while sargachromenol and sargaquinoic acid showed dose-dependent inhibitory activity against α-glucosidase (IC_50_ 42.41 ± 3.09 and 96.17 ± 3.48 µM, respectively). In the kinetic study of PTP1B enzyme inhibition, sargahydroquinoic acid and sargaquinoic acid led to mixed-type inhibition, whereas sargachromenol displayed noncompetitive-type inhibition. Moreover, plastoquinones dose-dependently inhibited ONOO^−^-mediated albumin nitration. Docking simulations of these plastoquinones demonstrated negative binding energies and close proximity to residues in the binding pocket of PTP1B and α-glucosidase, indicating that these plastoquinones have high affinity and tight binding capacity towards the active site of the enzymes. These results demonstrate that *S*. *serratifolium* and its major plastoquinones may have the potential as functional food ingredients for the prevention and treatment of type 2 diabetes.

## 1. Introduction

Diabetes mellitus (DM) has become a major growing public health problem worldwide. DM is a chronic disease that arises when the pancreas does not produce enough insulin or when the body cannot effectively use it, resulting in hyperglycemia. Type II DM (non-insulin-dependent diabetes) results from the ineffective use of insulin due to excess body weight, physical inactivity, and genetic susceptibility [1]. Protein tyrosine phosphatase 1B (PTP1B) is an intracellular phosphorylating enzyme, which controls both insulin and the leptin signaling pathway. PTP1B plays a prominent role in the regulation of cellular and metabolic processes [2]. PTP1B is highly expressed in tissues such as liver, fat and muscle, which are highly insulin targeted tissues [3]. The insulin-signaling pathway is a key pathway responsible for blood glucose regulation. PTP1B catalyzes the de-phosphorylation of activated insulin receptors, resulting in down-regulation of insulin signaling [4,5]. Furthermore, it has been reported that PTP1B knocked-out mice have resulted in enhanced insulin sensitivity, which indicate that PTP1B is the major player in the modulation of insulin sensitivity [6]. Nevertheless, a selective PTP1B inhibitor with an effective and safe pharmacological profile has not yet been identified [7].

Moreover, a well-established therapeutic approach to controlling postprandial hyperglycemia is inhibition of α-glucosidase, an enzyme that catalyzes the glucose release from the nonreducing end of dietary carbohydrates, thereby elevating blood glucose level. Inhibitors of α-glucosidase act in a competitive or non-competitive manner to inhibit α-glucosidase enzyme and lower the digestion of carbohydrates [8]. A previous report suggested that inhibition of this enzyme system helps to reduce the rate of carbohydrate digestion [9]. The existing clinical drugs used to target this enzyme, such as acarbose, miglitol, and voglibose, are associated with gastrointestinal side effects [10] and thus new α-glucosidase inhibitors without such side effects are needed for management of blood glucose. Moreover, nitrotyrosine is a product of ONOO^−^ action and the production of ONOO^−^ can be indirectly inferred by the presence of nitrotyrosine residues [11]. Tyrosine nitration by ONOO^−^ often leads to loss of protein activity thereby increasing the levels of nitrotyrosine as confirmed via the plasma of diabetic patients [12], while nitrotyrosine generation on the other hand was also found to alleviate acute glycemia and hyperglycemia [13]. Thus, PTP1B, α-glucosidase, and nitrotyrosine are promising targets in the development of new therapeutics for DM and other related metabolic syndromes.

*Sargassum* species are a group of large marine algae also known as macro brown algae or brown seaweed. It occurs globally and numerous coastal people have used it as a food source [14]. Among the different *Sargassum* spp., *Sargassum serratifolium* exists throughout the Korean and Japanese coastlines. Recently, many studies have shown that *S*. *serratifolium* extracts or compounds isolated from *S. serratifolium* have anti-microbial, anti-cancer, anti-inflammatory, and anti-Alzheimer’s disease effects [15,16,17,18,19]. However, the pharmacological effects of these extracts and the major constituents have not been studied thoroughly with regard to diabetes. Therefore, as part of our ongoing research to identify potent anti-diabetic agents from *S*. *serratifolium*, we isolated and investigated the activity of three plastoquinones (sargahydroquinoic acid, sargachromenol and sargaquinoic acid) against PTP1B, α-glucosidase, and ONOO^−^-mediated nitrated albumin. Enzyme kinetic analyses of these three plastoquinones against PTP1B were also performed using Dixon plots in order to confirm the type of enzymatic inhibition and to propose guidelines for the use of plastoquinones as anti-diabetic agents. Since no detailed information regarding the molecular interactions of sargahydroquinoic acid, sargachromenol and sargaquinoic acid with PTP1B and *α*-glucosidase is currently available, we performed molecular docking analysis to investigate their potential as anti-diabetic drug candidates. Here we report for the first time the potential of *S*. *serratifolium* and its major constituents to therapeutically treat DM.

## 2. Results

### 2.1. PTP1B and α-Glucosidase Inhibitory Activity of the EtOH Extract and the Solvent-Soluble Fractions of S. serratifolium

**Figure 1:**
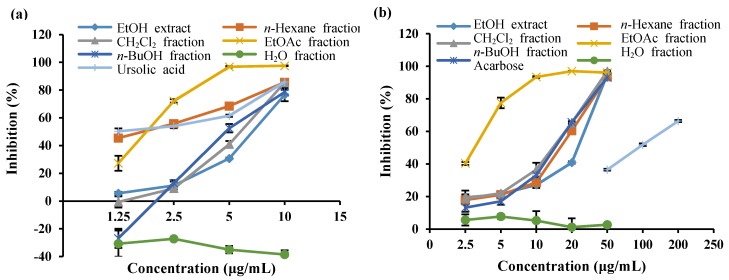
(**a**) PTP1B and (**b**) α-glucosidase inhibitory activity of the EtOH extract from *S. serratifolium* and its various fractions. Error bars indicate the standard error of the mean (SEM).

In order to evaluate the anti-diabetic potential of *S*. *serratifolium*, the EtOH extract and its fractions were tested via in vitro α-glucosidase and PTP1B inhibitory assays. The α-glucosidase and PTP1B inhibitory activity of the EtOH extract and its fractions are shown in Figure 1a,b and Table 1. The EtOH extract showed concentration-dependent inhibition against α-glucosidase and PTP1B with an IC_50_ (concentration required to decrease by 50%) of 24.16 ± 0.31 and 7.04 ± 0.26 μg/mL, respectively, compared to the positive controls acarbose (108.74 ± 2.96 μg/mL) and ursolic acid (1.12 ± 0.19 μg/mL). Since the EtOH extract of *S*. *serratifolium* showed both α-glucosidase and PTP1B inhibitory activity, it was further fractionated for detailed investigation. The EtOH extract of *S*. *serratifolium* was dissolved in H_2_O and successively partitioned with *n*-hexane, CH_2_Cl_2_, EtOAc, and *n*-BuOH to obtain different solvent-soluble fractions. The α-glucosidase and PTP1B inhibitory activity of the individual fractions of *S*. *serratifolium* was then evaluated. As shown in Table 1, the EtOAc fraction showed the highest α-glucosidase inhibitory activity, with an IC_50_ of 3.16 ± 0.10 μg/mL, which was significantly higher than that of the positive control acarbose (IC_50_ 108.74 ± 2.96 μg/mL). CH_2_Cl_2_, *n*-hexane, and *n-*BuOH fractions also showed potent α-glucosidase inhibitory potential with IC_50_ values of 14.61 ± 0.99, 16.73 ± 0.14, and 15.22 ± 0.25 μg/mL, respectively. However, the H_2_O fraction was found to be inactive on α-glucosidase inhibitory assay. As shown in Table 1, the *n*-hexane and EtOAc fractions exhibited the most PTP1B inhibitory potential, with IC_50_ values of 1.83 ± 0.06 and 1.88 ± 0.09 μg/mL, whereas the positive control, ursolic acid, had an IC_50_ value of 1.12 ± 0.19 μg/mL. In addition, both *n*-BuOH and CH_2_Cl_2_ fractions also showed significant PTP1B inhibitory activity, with IC_50_ values of 4.87 ± 0.24 and 6.32 ± 0.04 μg/mL, respectively. However, the H_2_O fraction was inactive, as observed in the PTP1B inhibitory assay.

### 2.2. Inhibitory Activity of Plastoquinones from S. serratifolium against PTP1B and α-Glucosidase

**Figure 2:**
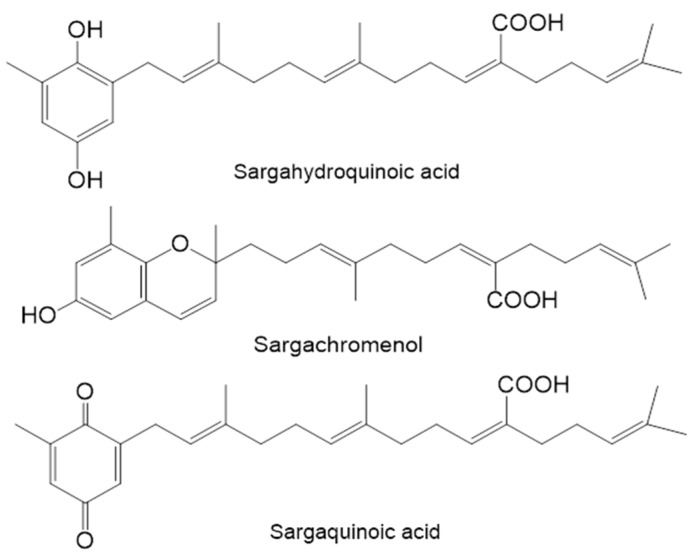
Chemical structures of the compounds isolated from *S. serratifolium*.

In order to evaluate the anti-diabetic activity of three plastoquinones (Figure 2) obtained from *S*. *serratifolium*, their inhibitory potential against PTP1B and α-glucosidase was evaluated using *p*NPP and *p*NPG as substrates, and the results are expressed as IC_50_ values (Table 2). Sargahydroquinoic acid showed the highest PTP1B inhibitory activity among the isolated compounds, with an IC_50_ of 5.14 ± 0.07 µM, which was significantly higher than the positive control, ursolic acid (IC_50_ 6.09 ± 0.02 µM). In addition, sargachromenol and sargaquinoic acid also exhibited potent PTP1B inhibitory activity with corresponding IC_50_ values of 11.80 ± 3.35 and 14.15 ± 0.02 µM, respectively. Among the tested compounds, sargachromenol exhibited promising α-glucosidase inhibitory activity with an IC_50_ value of 42.41 ± 3.09 µM compared to IC_50_ value of acarbose (210.76 ± 4.52 µM), while sargaquinoic acid also displayed potent α-glucosidase inhibitory activity with an IC_50_ value of 96.17 ± 3.48 µM. Interestingly, sargahydroquinoic acid was inactive as observed in the α-glucosidase inhibitory assay.

### 2.3. Enzyme Kinetics of PTP1B and α-Glucosidase Inhibition

**Figure 3:**
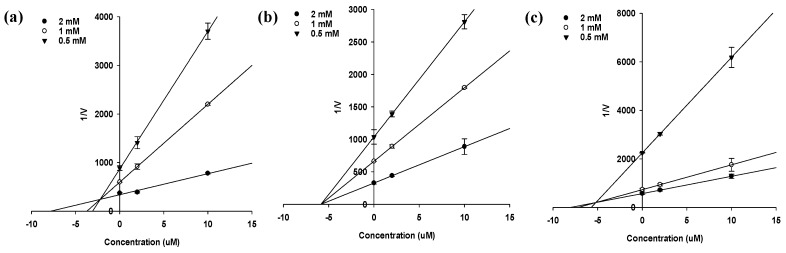
Dixon plots for PTP1B inhibition of compounds. (**a**) sargahydroquinoic acid; (**b**) sargachromenol and (**c**) sargaquinoic acid were tested in the presence of different concentrations of substrate: 2.0 mM (●); 1.0 mM (ο); 0.5 mM (▼).

**Figure 4:**
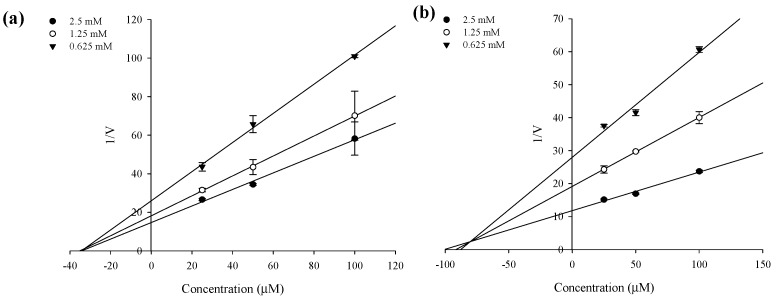
Dixon plots for α-glucosidase inhibition of compounds. (**a**) sargachromenol and (**b**) sargaquinoic acid were tested in the presence of different concentrations of substrate: 2.5 mM (●); 1.25 mM (ο); 0.625 mM (▼).

In an attempt to explain the mode of enzymatic inhibition of sargahydroquinoic acid, sargachromenol, and sargaquinoic acid, kinetic analysis was performed at different concentrations of the substrate (*p*NPP for PTP1B and *p*NPG for α-glucosidase) and inhibitor. Dixon plots are a graphical method [a plot of 1/enzyme velocity (1/*V*) against inhibitor concentration (I)] of determining the type of enzyme inhibition and dissociation constant (*K_i_*) of an enzyme-inhibitor complex. Figure 3 and Figure 4 and Table 2 demonstrate the enzymatic kinetic results of sargahydroquinoic acid, sargachromenol and sargaquinoic acid. Sargahydroquinoic acid and sargaquinoic acid exhibited mixed-type inhibition, while sargachromenol displayed noncompetitive-type PTP1B inhibition (Figure 3a–c) with respective *K_i_* values of 2.21, 5.20, and 5.85 μM, respectively (Table 2), and sargachromenol and sarquinoic acid showed noncompetitive type and mixed-type α-glucosidase inhibition (Figure 4a,b) with *K_i_* values of 33.95 and 79.68 μM (Table 2), accordingly. As the *K_i_* value represents the concentration needed to form an enzyme-inhibitor complex, a lower *K_i_* value may indicate more effective inhibition against PTP1B in the development of preventive and therapeutic agents.

### 2.4. Inhibitory Effect of Plastoquinones on ONOO^–^-Mediated Albumin Nitration

**Figure 5:**
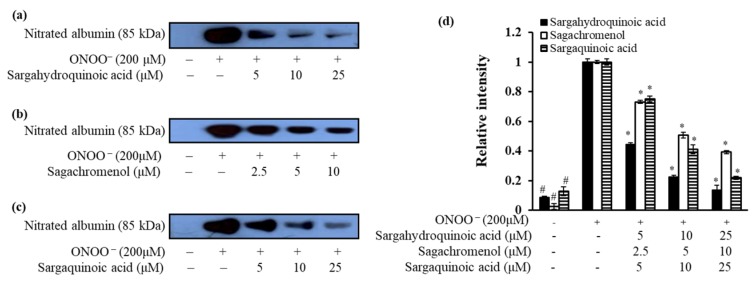
Dose-depended inhibition of ONOO^−^-mediated albumin nitration by plastoquinones. Mixtures of test samples, bovine serum albumin (BSA), and ONOO^−^ were incubated with shaking at 37 °C for 30 min. The reactant was resolved in 10% polyacrylamide gel via electrophoresis. (**a**) sargahydroquinoic acid; (**b**) sargachromenol; and (**c**) sargaquinoic acid were used at the indicated concentrations; (**d**) Quantification of band intensity was calculated using CS Analyzer 3.00 (ATTO Corp., Tokyo, Japan). ^#^ *p* < 0.05 indicates a significant difference from the untreated normal group, * *p* < 0.05 indicate significant differences from the ONOO^−^ treated control.

To evaluate the inhibitory effect of these plastoquinones against ONOO^–^-induced nitration of albumin, western blot analysis was performed using 3-nitrotyrosine antibody. As shown in Figure 5b, pretreatment with sargachromenol at different concentrations (2.5–10 μM) resulted in strong inhibition of ONOO^–^-mediated albumin nitration in a concentration-dependent manner. Moreover, pretreatment with sargahydroquinoic acid and sargaquinoic acid at different concentrations (5–25 μM) resulted in markedly dose-dependent inhibition of ONOO^−^-mediated nitration of albumin (Figure 5).

### 2.5. Molecular Docking Simulation of PTP1B Inhibition

**Figure 6:**
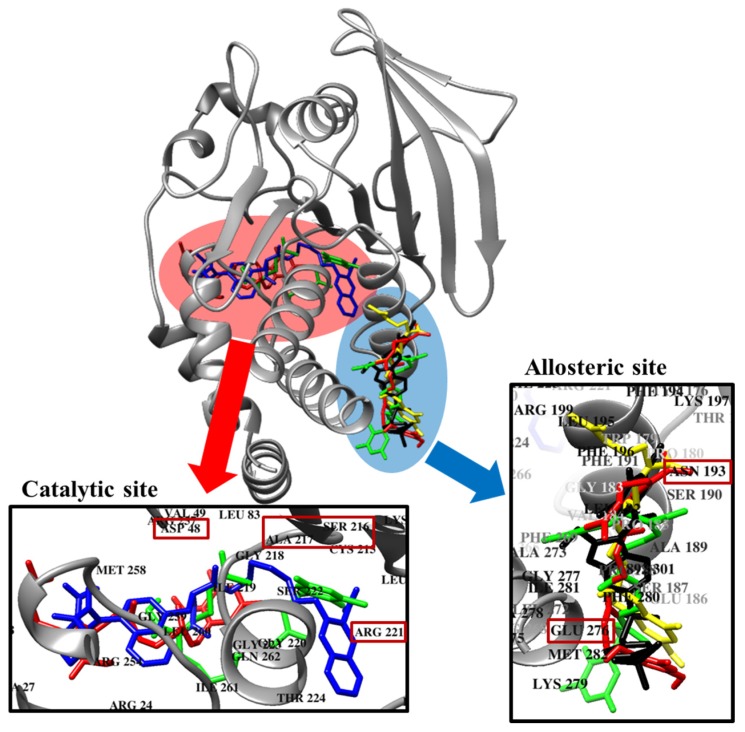
Molecular docking related PTP1B inhibition by compound **23**, compound **2**, sargahydroquinoic acid, sargachromenol and sargaquinoic acid. Binding sites of compound **23**, compound **2**, sargahydroquinoic acid, sargachromenol and sargaquinoic acid are represented by *blue*, *black*, *red*, *yellow* and *green* structures, respectively.

**Figure 7:**
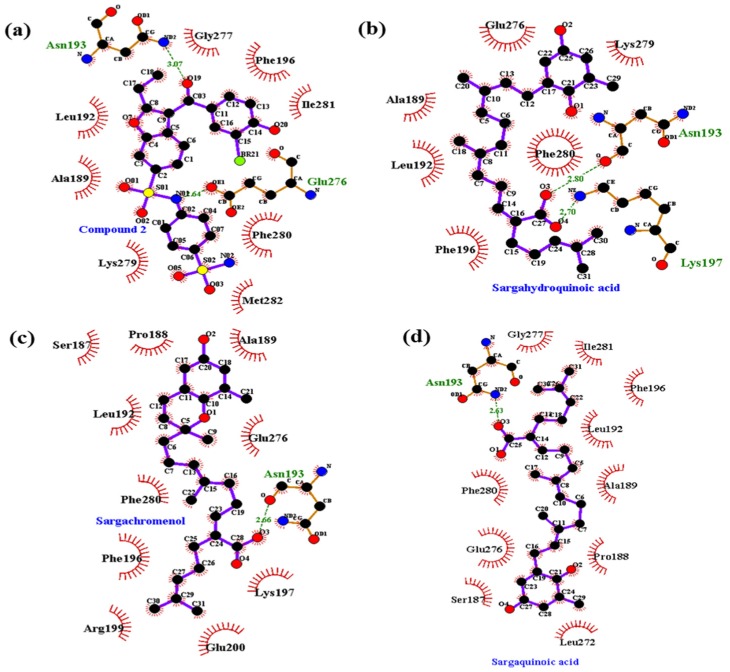
Molecular docking models for PTP1B allosteric inhibition of (**a**) compound **2**; (**b**) sargahydroquinoic acid; (**c**) sargachromenol and (**d**) sargaquinoic acid.

**Figure 8:**
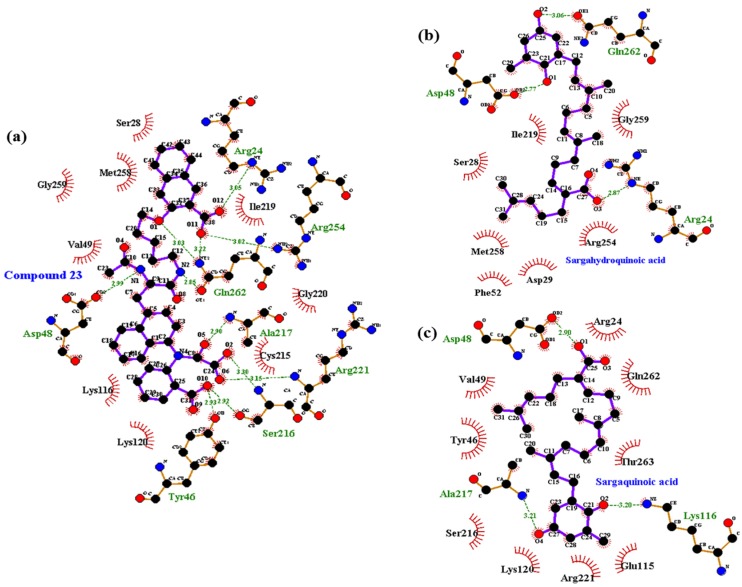
Molecular docking models for PTP1B catalytic inhibition of (**a**) compound **23**; (**b**) sargahydroquinoic acid and (**c**) sargaquinoic acid.

Molecular docking simulation is a good option for investigating protein-ligand interaction geometries at the molecular level. In these studies, molecular docking simulation of sargahydroquinoic acid, sargachromenol, and sargaquinoic acid with PTP1B was performed, where 3-({5-[(*N*-acetyl-3-{4-[(carboxycarbonyl)(2-carboxyphenyl)amino]-1-naphthyl}-l-alanyl)amino] pentyl}oxy)-2-naphthoic acid (compound **23**) and (3-(3,5-dibromo-4-hydroxy-benzoyl)-2-ethyl-benzofuran-6-sulfonic acid 4-sulfamoyl-phenyl)-amide (compound **2**) were considered the standard ligand for validating the AutoDock 4.2 results (Figure 6). The binding energies of sargahydroquinoic acid, sargachromenol, and sargaquinoic acid with interacting residues including H-bond interacting residues and van der Waals interacting residues along with the number of H-bonds are listed in Table 3. The simulation results of AutoDock 4.2 are shown in Figure 6 and the PTP1B-sargahydroquinoic acid inhibitor complex showed a −5.95 kcal/mol binding energy with two hydrogen bonds and interacting residues of Asn193 and Lys197. As illustrated in Figure 7b, the corresponding ligand interactions of sargahydroquinoic acid at the active site of PTP1B are the two hydrogen-bonding interactions between the Asn193 and Lys197 residues of the enzyme with the carboxylic group of sargahydroquinoic acid and bond distances of 2.80 and 2.70 Å, respectively. In addition, hydrophobic interactions were also observed between Ala189, Leu192, Phe196, Glu276, Lys279, and Phe280. In addition, sargahydroquinoic acid exhibited catalytic inhibition against PTP1B displaying three H-bonds with the residues Arg24, Asp48 and Gln262, and hydrophobic interactions between Ser28, Asp29, Phe52, Ile219, Arg254, Met258 and Gly259 (Figure 8b). As illustrated in Figure 7c, sargachromenol exhibited a −8.84 kcal/mol binding affinity to PTP1B and also bound to PTP1B via the formation of a hydrogen bond, as shown in Table 3. The binding of sargachromenol involved the formation of a hydrogen bond between the Asn193 residue of PTP1B and the interacting sargachromenol carboxylic group. In addition, the residues Ser187, Pro188, Ala189, Leu192, Phe196, Lys197, Arg199, Glu200, Glu276, and Phe280 formed hydrophobic interactions, thereby strengthening the protein-ligand interaction between PTP1B and sargachromenol. As illustrated in Figure 7d, sargaquinoic acid exhibited a −6.83 kcal/mol binding affinity to allosteric site of PTP1B. Additionally, the interacting carboxylic group exhibited a hydrogen bond interaction with the Asn193 residue. Hydrophobic interactions were observed between sargaquinoic acid and PTP1B residues Ser187, Pro188, Ala189, Leu192, Phe196, Leu272, Glu276, Gly277, Phe280, and Ile281 further stabilized the protein-ligand interaction. Additionally, sargaquinoic acid bound to catalytic site of PTP1B showing weak binding affinity (−3.13 kcal/mol) illustrating three H-bonds with Asp48, Lys116 and Ala217, and van der Waals bonds between Arg24, Tyr46, Val49, Glu115, Lys120, Ser216, Arg221, Gln262 and Thr263 (Figure 8c). Therefore, these results of the docking simulation support the results of enzyme kinetics. On the other hand, the Arg24, Tyr46, Asp48, Ser216, Ala217, Arg221, Arg254, and Gln262 enzyme residues participated in hydrogen-bonding interactions with compound **23**, and the residues of Asn193 and Glu276 participated in hydrogen-bonding interactions with compound **2**. Moreover, the binding energies of both compounds were negative (−11.23 kcal/mol for compound **23** and −10.98 kcal/mol for compound **2**), indicating that additional hydrogen bonding might stabilize the open form of the enzyme and potentiate tighter binding to the PTP1B active site, resulting in more effective PTP1B inhibition.

### 2.6. Molecular Docking Simulation of α-Glucosidase Inhibition

**Figure 9:**
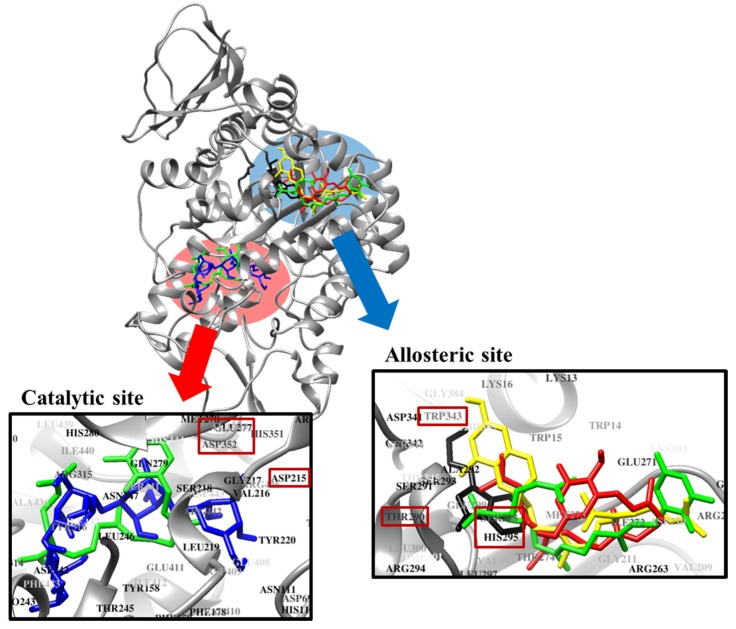
Molecular docking related to α-glucosidase inhibition by acarbose, BIP, sargahydroquinoic acid, sargachromenol and sargaquinoic acid. Binding sites of acarbose, BIP, sargahydroquinoic acid, sargachromenol and sargaquinoic acid are represented by *blue*, *black*, *red*, *yellow* and *green* colored structures, respectively.

**Figure 10:**
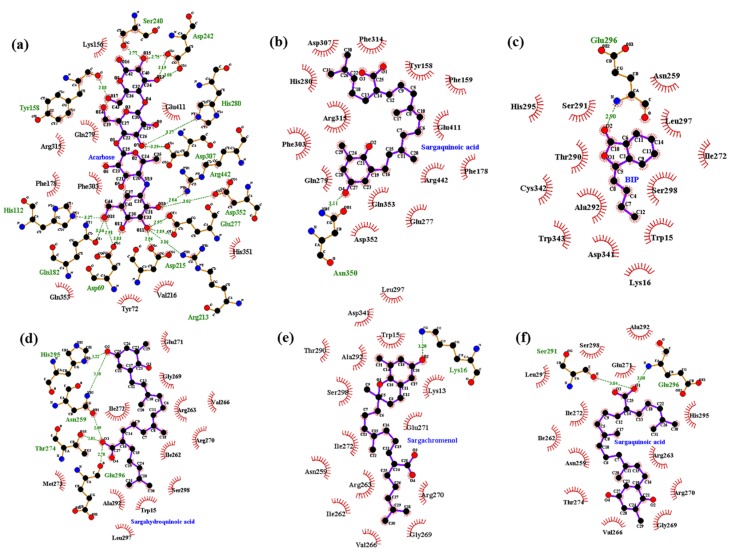
Molecular docking models for α-glucosidase inhibition by (**a**) acarbose; (**b**) sargaquinoic acid; (**c**) BIP; (**d**) sargahydroquinoic acid; (**e**) sargachromenol and (**f**) sargaquinoic acid.

Molecular docking simulation of sargahydroquinoic acid, sargachromenol, and sargaquinoic acid with α-glucosidase was performed, and the ligand–enzyme complexes of the three plastoquinones/or acarbose and (*Z*)-3-butylidenephthalide (BIP) were stably posed in the same pocket of the α-glucosidases by AutoDock 4.2 (Figure 9). The binding energies of sargahydroquinoic acid, sargachromenol, and sargaquinoic acid with interacting residues, including H-bond interacting residues and van der Waals interacting residues, along with the number of H-bonds, are listed in Table 4. According to the AutoDock 4.2 simulation result shown in Figure 10d, the α-glucosidase-sargahydroquinoic acid inhibitor complex showed −8.0 kcal/mol binding energy containing five hydrogen bonds with interacting residues Glu296, Asn259, Thr274, and His295. In addition, hydrophobic interactions were also observed between Trp15, Ile262, Arg270, Ile272, Val266, Ala292, Met273, Leu297, Ser298, Gly269, Glu271, and Arg263 residues. Moreover, sargachromenol binding affinity with α-glucosidase was −7.3 kcal/mol for a hydrogen bond with interacting Lys16 residue. In addition, some hydrophobic interactions were involved with the Lys13, Trp15, Asn259, Ile262, Arg263, Val266, Gly269, Arg270, Glu271, Ile272, Thr290, Ala292, Leu297, Ser298, and Asp341 residues (Figure 10e). In contrast, the α-glucosidase-sargaquinoic acid inhibitor complex showed −7.1 kcal/mol binding energy for two hydrogen bonds with interacting residues Ser291 and Glu296 in allosteric site of α-glucosidase. In addition, hydrophobic interactions were also observed between Asn259, Ile262, Arg263, Val266, Gly269, Arg270, Glu271, Ile272, Thr274, Leu297, Ala292, His295, and Ser298 residues (Figure 10f). Furthermore, some residues of the catalytic site interact with sargaquinoic acid displaying a hydrogen bond and hydrophobic interactions (Figure 10b). As shown in Figure 10a, the docking results of a known α-glucosidase catalytic inhibitor acarbose formed seventeen hydrogen bonds with interacting Asp69, Gln82, His112, Tyr158, Arg213, Asp215, Ser240, Asp242, Glu277, His280, Asp307, Asp352, and Arg442 residues, whereas BIP is a potent α-glucosidase allosteric inhibitor and formed a hydrogen bond with interacting Glu296 residue (Figure 10c).

## 3. Discussion

Diabetes is a chronic degenerative metabolic disease with high morbidity and mortality rates due to associated complications. Because of the increasing number of diabetic patients and the limited number of anti-diabetic drugs available, the search for new compounds, especially from marine sources, has attracted a great deal of interest from the scientific community. Seaweeds are a common food source consumed around the world [20,21]. Seaweeds are sources of bioactive compounds with immense medicinal potential that have attracted the attention of pharmaceutical industries [22,23]. Korean marine macro algae exhibit rich diversity because of the varied habitats available and mixing of warm and cold currents. Approximately 870 macro algal species have been reported in Korea [24]. The genus *Sargassum* is widely distributed in temperate and tropical oceans. Many studies have sought to determine the bioactive compounds produced by marine algae. Brown algae of the genus *Sargassum* contain structurally unique secondary metabolites such as plastoquinones, chromanols, polysaccharides, fucoidans, phlorotannins, and phytosterols [20,21]. There have been numerous reports on their secondary metabolites and related biological activities, such as cell toxicity, antioxidant activity, vasodilatory effects, induction of hydrozoan larval settlement and inhibition of acetylcholinesterase [23,25,26,27,28]. Previously, Laminaria and Ecklonia species were administered to patients with diabetes mellitus in an interesting application of marine natural products [21]. There are many active compounds that could be useful for managing diabetes mellitus [20,21]. For example, fucoidan, a newly detected α-D-glucosidase inhibitor from S. wightii, might be useful in type 2 diabetes mellitus therapy [29]. Thunberol is another new sterol isolated from the Chinese brown alga *S. thunbergii* [30]. D’Orazio et al. [31] found that fucoxanthin, a characteristic carotenoid present in brown seaweeds, prevented the development of diabetes through down-regulation of inflammatory mediator mRNA levels in a mouse model of obesity/diabetes, as well as promoted the recovery of blood glucose uptake by muscle via the up-regulation of glucose transporter 4. In addition, some phlorotannins isolated from brown algae, *Eisenia,* Ecklonia and *Ishige* species such as phlorofucofuroeckol-A, dieckol, eckol, and diphlorethohydroxycarmalol exhibited anti-diabetic activity [32,33,34,35].

*Sargassum serratifolium* C. Agardh (Phaeophyceae, Fucales) possesses anti-tumor [36] and anti-cancer [17] activities. Previous phytochemical investigations isolated plastoquinones as secondary metabolites, including sargaquinoic acid, sargahydrogquinoic acid, sargaquinal, and sargachromenol [37], which possess a wide range of biological activities, including anti-cholinesterase [26], anti-hyperproliferative disease [38], neuro-protective [39,40], photo-protective [41], anti-inflammatory [42,43,44], age-related inflammation disease and skin aging protection [45], anti-diabetic and hypolipidemic [46], anti-vascular inflammatory [19], anti-adipogenic [47], and anti-carcinogenic [48] properties. Nevertheless, a *S. serratifolium* preparation has not yet been applied to human health until now, which led us to demonstrate the anti-diabetic potential of *S. serratifolium* extract and its three major compounds through the inhibition of PTP1B and α-glucosidase. PTP1B is involved in cell proliferation, survival and migration, cytoskeletal organization, cell–cell communication, metabolism and energy expenditure [49,50,51]. In addition, PTP1B dephosphorylates specific phosphotyrosine (pTyr) residues of the activated insulin receptor and IRS proteins, thus interrupting insulin signaling and intervening glucose homeostasis [52,53]. In addition, PTP1B downregulates the leptin signaling by dephosphorylating JAK2, a kinase associated with the leptin receptor, which is crucial in the recruitment and activation of signal transducer and activator of transcription 3 and the consequent transcription of genes involved in feeding and energy homeostasis [54,55].

Despite a number of drug candidates of both natural and synthetic origin, no PTP1B inhibitors have been clinically approved [56,57]. This might be because most of the PTP1B inhibitors discovered thus far are highly charged molecules, which limits their potential clinical use [58]. The purpose of this study was to identify new PTP1B inhibitors from *S. serratifolium* for the treatment of diabetes. We found that the EtOH extract of *S. serratifolium* inhibited PTP1B. Consequently, the EtOH extract of the *S. serratifolium* was further partitioned by systematic fractionation. Of the tested fractions, the *n*-hexane and EtOAc fractions potently inhibited PTP1B, with IC_50_ values of 1.83 ± 0.06 and 1.88 ± 0.09 µg/mL, respectively. In comparison, the IC_50_ value of ursolic acid, a well-known PTP1B inhibitor, was 2.78 ± 0.02 µg/mL. Therefore, we focused on isolating PTP1B inhibitor compounds from the *n*-hexane fraction due to the high yield and potent activity compared to other fractions. We isolated three plastoquinones, all of which significantly inhibited PTP1B. Sargahydrogquinoic acid, sargachromenol, and sargaquinoic acid were potent inhibitors of PTP1B with IC_50_ values of 5.14 ± 0.07, 11.80 ± 3.35, and 14.15 ± 0.02 µM. Furthermore, we also investigated inhibition type using an enzyme kinetic study in the presence of varying substrate and inhibitor concentrations. These studies revealed that sargahydrogquinoic acid and sargaquinoic acid exhibited mixed-type PTP1B inhibition, indicating that these compounds can bind to the allosteric site of the free enzyme or to the enzyme-substrate complex, whereas sargachromenol showed noncompetitive inhibition, with binding to the enzyme-substrate complex.

In order to confirm the inhibition mode of the PTP1B enzyme, we predicted the 3D structure of PTP1B using AutoDock 4.2 to simulate the binding of three plastoquinones and compound **23**, a PTP1B enzyme inhibitor [59]. Compound **23** is among the most potent non-peptic PTP1B catalytic inhibitor reported to date. Moreover, we also predicted the 3D structure of PTP1B using AutoDock 4.2 to simulate the binding of these plastoquinones and compound **2**, a PTP1B allosteric inhibitor [7]. The AutoDock 4.2 docking program was used to predict protein–ligand binding interactions. Currently, automated docking is widely used as an effective means of quick and accurate prediction of biomolecular conformation and binding energy of protein–ligand complexes in molecular design. The molecular docking models of sargahydrogquinoic acid, sargachromenol, sargaquinoic acid, compound **23**, and compound **2** are illustrated in Figure 6. Ligand–enzyme complexes with three plastoquinones/or compound **2** were stably posed in the same pocket of PTP1B by AutoDock 4.2. As illustrated in Figure 7b, in the corresponding ligand interactions of sargahydrogquinoic acid at the active site of PTP1B, there are two hydrogen-bonding interactions with two important residues (Asn193 COOH and Lys197 COOH) of the enzyme, and sargaquinoic acid also bound at catalytic site of PTP1B (Figure 8c). In the case of sargachromenol, an important residue of the enzyme (Asn193 COOH) participated in the hydrogen-bonding interaction (Figure 7c). Moreover, an important residue of the enzyme (Asn193 COOH) participated in the hydrogen-bonding interaction and residues of allosteric site of PTP1B interacted with sargaquinoic acid (Figure 7d). Additionally, H-bonds are formed between the three compounds and the key Asn193 residue stabilizes these compounds and allows them to fit together at the enzyme allosteric site. Wiesmann et al. [7] also reported that the Ans193 residue in hydrogen bond formation is important for PTP1B allosteric inhibitors. Our results were in accordance with Wiesmann et al. [7], who suggested that the hydrogen bond formation with Asn193 is the minimum requirement for any compound to be considered an allosteric PTP1B inhibitor. In addition, the binding energy of plastoquinones was negative, indicating that hydrogen bonding may stabilize the open form of the enzyme and potentiate tight binding of the active site of PTP1B, resulting in more effective PTP1B inhibition. Taken together, the results of kinetic analysis and molecular docking simulation indicate that these three plastoquinones have promising anti-diabetic properties.

Mammalian α-glucosidases, an exo type of carbohydrase located in the brush-border surface membrane of intestinal cells, is a key enzyme that catalyzes the final step in the digestive process of carbohydrates. The pharmaceutical research community is interested in α-glucosidase because inhibition of this enzyme delays carbohydrate digestion, causing a reduction in the rate of glucose absorption and decreases postprandial blood glucose. Thus, effective α-glucosidase inhibitors may be promising chemotherapeutic agents for clinical use in the treatment of diabetes [60,61].

Enzyme inhibition is an important tool in pharmaceutical research as well as in the field of drug discovery. In the present study, we found that the 70% EtOH extract of *S. serratifolium* and its solvent-soluble fractions exhibited dose-dependent α-glucosidase inhibitory activity. Moreover, we isolated the active compounds effective against α-glucosidase from *S. serratifolium*, with sargachromenol and sargaquinoic acid (IC_50_ = 42.41 and 96.17 µM, respectively) showing potent inhibition superior to the standard α-glucosidase inhibitor. Although the 3D structures of some bacterial α-glucosidase have been reported, the X-ray crystal structure information of α-glucosidase was still unknown. However, the potential of some inhibitors greatly depends on the enzyme’s origin, especially on yeast or mammalian α-glucosidase [62]. Oku et al. [63] reported that rat intestinal enzymes could be correlated to estimate the activity of human enzymes. Therefore, further study needs to be undertaken to confirm our findings via in vivo experiments.

In addition, a molecular docking study was carried out to explore the binding mode of plastoquinones within the binding pocket of α-glucosidase and to understand their molecular interactions using AutoDock 4.2. As a means of testing the adopted protocols, known inhibitor acarbose (the first α-glucosidase inhibitor approved for type 2 diabetes treatment) was docked into the binding pocket of a developed homology model. Acarbose fit well in the binding pocket and showed interaction with important active site residues. All three plastoquinones were docked in the binding pocket of a developed homology model of α-glucosidase enzyme. Based on molecular docking, the top-ranked conformation of sargahydroquinoic acid and sargaquinoic acid was five hydrogen bonds between the carboxylic group on the linear terpene chain moiety of the compound and the active site residues (Glu296, Asn259, Thr274, His295, and Ser291). The hydroxyl group of sargahydroquinoic acid and sargachromenol also exhibited hydrogen interactions with the His295, Asn259, and Lys16 residues. Additionally, H-bonds were formed between three compounds and the key Glu296, Thr274, His295, and Ser291 residues stabilized these compounds at the active site and allowed these compounds to fit in the enzyme active pocket. Furthermore, several hydrophobic interactions were observed between compounds and active site residues (Ser298, Gly269, His295, Thr290, Arg263, Val266, Gly269, and Ala292) that stabilized the binding of these compounds at the active site of α-glucosidase. Together, these results suggest that these three plastoquinones do not bind to the same catalytic site in α-glucosidase as acarbose. Furthermore, the allosteric inhibitor BIP and the three plastoquinones exhibited similar binding residues (Trp15, Lys16, Asn259, Glu296, His295, and Ala292), which indicates that these three plastoquinones may act as allosteric inhibitors against α-glucosidase. Indeed, docking analysis suggested that all three plastoquinones bind to the enzyme in a different pocket than acarbose. So, the docking study was validated with the allosteric inhibitor, BIP. Interestingly, the decomposition of peroxynitrite will form highly reactive species leading to the oxidation of cysteines in proteins. This is of particular interest with regard to PTP1B where S-nitrosation was shown to inactivate the enzyme [64]. In addition, the prenylated quinone, plastoquinones were postulated to play a part in the regulation of gene expression and signal transduction within cells because of its high hydrophobicity [65]. Although the plastoquinones were found to display various biological activities via in vitro assays, their overall in vivo function is yet to be clarified. Since these plastoquinones are structurally related to vitamin E, they are also prone to exhibit similar antioxidative and anti-cancerous activities [65]. Recently, some in vivo studies revealed that plastoquinones in *Sargassum* sp. could be employed in the treatment of hyperproliferative skin diseases, gastric ulcer, and cerebral vascular diseases [38,66,67]. Thus, these three plastoquinones isolated from *S. serratifolium* could act as a dual PTP1B/α-glucosidases inhibitors and peroxinitrate-mediated nitration of albumin scavenger.

## 4. Materials and Methods

### 4.1. General Experimental Procedures

^1^H- and ^13^C-NMR spectra were obtained using a JEOL JNM ECP-400 spectrometer (Tokyo, Japan) at 400 MHz for ^1^H and 100 MHz for ^13^C in deuterated chloroform (CDCl_3_). The high-performance liquid chromatography–quadrupole-time-of-flight tandem mass spectrometry (HPLC-Q-TOF-MS) analysis was performed with a 1260 Infinity HPLC system (Agilent Corp., Santa Clara, CA, USA) with Impact HD-Q-TOF Mass spectrometry (Bruker Corp., Bremen, Germany). Column chromatography was performed using silica gel 60 (70–230 mesh, Merck, Darmstadt, Germany), and LiChroprep RP-18 (40–63 µm, Merck). All TLC analyses used precoated Merck Kieselgel 60 F_254_ plates (20 × 20 cm, 0.25 mm, Merck) and 50% H_2_SO_4_ as the spray reagent.

### 4.2. Chemicals and Reagents

*p*-Nitrophenyl α-d-glucopyranoside (*p*NPG), acarbose, *p*-nitrophenyl phosphate (*p*NPP), Yeast α-glucosidase, and ethylenediaminetetraacetic acid (EDTA) were purchased from Sigma-Aldrich. Dithiothreitol (DTT) was purchased from Bio-Rad Laboratories (Hercules, CA, USA) and PTP1B (human recombinant) was purchased from Biomol International LP (Plymouth Meeting, PA, USA). ONOO^−^ was purchased from Molecular Probes Cayman (Ann Arbor, MI, USA). All other chemicals and solvents used were purchased from E. Merck, Fluka, and Sigma-Aldrich, unless otherwise stated.

### 4.3. Extraction, Fractionation, and Isolation

*Sargassum serratifolium* was collected along the coast of Busan, South Korea in April 2014. Specimen identity was confirmed by an algal biologist (N. G. Kim) at the Department of Marine Biology and Aquaculture, Gyeongsang National University, South Korea. Dried seaweed (3.5 kg) was extracted twice with 95% (*v*/*v*) ethanol (6 L/each) at 70 °C for 3 h. The ethanolic extract (250.3 g) was obtained after concentration under reduced pressure and was then suspended in water (3:1). The suspension was successively partitioned with *n*-hexane, CH_2_Cl_2_, EtOAc, and *n*-BuOH to yield the *n*-hexane (86.5 g), CH_2_Cl_2_ (36 g), EtOAc (22.6 g), and *n*-BuOH (37.2 g) fractions, respectively, as well as the H_2_O residue (55.3 g). Sargahydroquinoic acid, sargachromenol and sargaquinoic acid were isolated from *n*-hexane fraction according to the method described by Joung et al. [17], and identified by direct comparison with authentic samples (^1^H- and ^13^C-NMR). The purity of all compounds was determined to be higher than 99% by HPLC-Q-TOF-MS analysis. The structures of these compounds are shown in Figure 2.

### 4.4. PTP1B Inhibitory Assay

The PTP1B inhibitory activities of the plastoquinones were evaluated using *p*NPP [68]. Recombinant PTP1B enzyme (0.5 units diluted with a PTP1B reaction buffer) was added with or without sample. The plate was pre-incubated at 37 °C for 10 min and then substrate (2 mM *p*NPP) was added. Following incubation at 37 °C for 15 min, the enzymatic reaction was terminated by the addition of 10 M NaOH. The absorbance was measured at 405 nm using a microplate spectrophotometer (Molecular Devices, Sunnyvale, CA, USA). Ursolic acid was used as a standard compound.

### 4.5. α-Glucosidase Inhibitory Assay

The enzyme inhibition study was carried out spectrophotometrically using the procedure reported by Li et al. [69]. The α-glucosidase activity was determined by measuring release of *p*NPG at 405 nm using a microplate spectrophotometer (Molecular Devices). Acarbose was used as a standard compound.

### 4.6. Inhibition of ONOO^–^-Mediated Albumin Nitration

The inhibition of ONOO^–^-mediated nitrated albumin was evaluated using the method of Aulak et al. [70]. Samples were added to bovine serum albumin (BSA) and mixed with ONOO^−^ (200 µM). After 10 min incubation (room temperature), the sample solution was added to Bio-Rad 2X Laemmli Sample buffer with mercaptoethanol and then boiled for 7 min. The reactant was resolved in 10% polyacrylamide gel via electrophoresis and transferred onto PVDF membranes. Pre-stained blue protein markers were used for molecular weight determination. Monoclonal anti-nitrotyrosine antibody (85 kDa) was used as a primary antibody and horseradish peroxidase-conjugated sheep anti-mouse secondary antibody was used as a secondary antibody. Antibody labeling was visualized using the Supersignal West Pico Chemiluminescent substrate (Pierce Chemical Co., Rockford, IL, USA). Densitometric analysis of the western blot results was determined using CS analyzer v3.00 (ATTO Corp.).

### 4.7. Kinetic Study of Plastoquinones against PTP1B and α-Glucosidase

To determine the modes of enzyme inhibition, Dixon plots were used [71]. The kinetic analyses were performed at different concentrations of substrate (0.625, 1.25, and 2.5 mM *p*NPG for α-glucosidase; 0.5, 1.0, and 2.0 mM *p*NPP for PTP1B) and various concentrations of plastoquinones (0, 0.4, 2.0, and 10.0 µM for PTP1B and 0, 25, 50 and 100 µM for α-glucosidase, respectively). Enzymatic inhibition of the test plastoquinones was evaluated by monitoring the effects of different concentrations of the substrate in the Dixon plots (single reciprocal plots). The enzymatic procedure consisted of the same aforementioned assay method. The inhibition constants (*K_i_*) were determined via interpretation of the Dixon plots, where the value of the *x*-axis implies -*K_i_* [71,72].

### 4.8. Molecular Docking Simulation of PTP1B and α-Glucosidase Inhibition

The structure of PTP1B, with its selective allosteric inhibitor 3-(3,5-dibromo-4-hydroxy-benzoyl)-2-ethyl-benzofuran-6-sulfonic acid (4-sulfamoyl-phenyl)-amide (compound **2**) (PDB ID: 1T49) and 3D structure of catalytic inhibitor 3-({5-[(*N*-acetyl-3-{4-[(carboxycarbonyl)(2-carboxyphenyl)amino]-1-naphthyl}-l-alanyl)amino]pentyl}oxy)-2-naphthoic acid (compound **23**) were obtained from the RCSB Protein Data Bank website [7] and PubChem Compound (NCBI) with a compound CID of 447410, respectively. In contrast, the structure of α-glucosidase, with its catalytic ligand α-d-glucose (PDB ID: 3A4A) and the structure of acarbose and (*Z*)-3-butylidenephthalide (BIP) were obtained from the RCSB Protein Data Bank website [73] and PubChem Compound (NCBI) with compound CIDs of 41,774 and 5,352,899, respectively. Protein preparation was conducted using Accelrys Discovery Studio 16.1 (Accelrys, Inc., San Diego, CA, USA). The binding areas of compound **23**, compound **2**, acarbose, and BIP of the protein were considered to be the most convenient regions for ligand binding in the docking simulation. The 3D structures of sargahydroquinoic acid, sargachromenol and sargaquinoic acid were obtained from PubChem Compound (NCBI) and protonated using the MarvinSketch (ChemAxon, Budapest, Hungary). A Lamarckian genetic algorithm (GA) method was used for docking. Gasteiger charges were added by default, the rotatable bonds were set with ADT, and all torsions were allowed to rotate. Grid box size was set to maximum with a default spacing. The X, Y, Z center was 37.303, 30.97, 33.501 for PTP1B and 21.272, −0.751, 18.633 for α-glucosidase. The docking simulation was conducted 10 independent GA with the default parameters. The results were analyzed using UCSF Chimera (http://www.cgl.ucsf.edu/chimera/), while the hydrogen bond interacting residues and van der waals interacting residues were visualized using LigPlot^+^.

### 4.9. Statistics

All results are expressed as the mean ± SEM of triplicate samples. Statistical significance was analyzed using one-way ANOVA and Duncan’s test (Systat Inc., Evanston, IL, USA), and was noted at *p* < 0.05.

## 5. Conclusions

In conclusion, our results demonstrated that *S. serratifolium* and its constituents may have the potential for therapeutic treatment of DM through PTP1B and α-glucosidase inhibition. Of the three isolated plastoquinones, sargahydroquinoic acid showed potent PTP1B inhibitory activity, while sargachromenol and sargaquinoic acid showed potent α-glucosidase inhibitory activity. These plastoquinones also effectively suppressed ONOO^−^-mediated albumin nitration in a dose-dependent manner. In addition, enzyme kinetics analysis of the type of enzyme inhibition and molecular docking simulation between enzymes and active compounds supported the above results. Therefore, *S. serratifolium* and its constituents should be further explored for the development of novel therapeutic or preventive agents for the treatment of diabetes.

## Figures and Tables

**Table 1:** Protein tyrosine phosphatase 1B and α-glucosidase inhibitory activity of the EtOH extract from *S. serratifolium* and its various fractions.

Sample	IC_50_ Values (Mean ± SEM) ^a^	
	PTP1B	α-Glucosidase
EtOH extract	7.04 ± 0.26 ^d^	24.16 ± 0.31 ^e^
*n*-Hexane fraction	1.83 ± 0.06 ^g^	16.73 ± 0.14 ^f^
CH_2_Cl_2_ fraction	6.32 ± 0.04 ^e^	14.61 ± 0.99 ^g^
EtOAc fraction	1.88 ± 0.09 ^g^	3.16 ± 0.10 ^h^
*n*-BuOH fraction	4.87 ± 0.24 ^f^	15.22 ± 0.25 ^g^
H_2_O fraction	>100	>100
Ursolic acid ^b^	1.12 ± 0.19 ^h^	
Acarbose ^c^		108.74 ± 2.96 ^d^

^a^ The 50% inhibitory concentrations (IC_50_, µg/mL) are expressed as the mean ± SEM of three experiments; ^b,c^ Positive controls for the PTP1B and α-glucosidase assays, respectively; ^d–h^ Mean with different letters are significantly different with Duncan’s test at *p* < 0.05.

**Table 2:** Protein tyrosine phosphatase 1B and α-glucosidase inhibitory activity of the compounds isolated from *S. serratifolium.*

Compound	PTP1B			α-Glucosidase		
	IC_50_ (μM) ^a^	Inhibition Type ^b^	*K_i_* (μM) ^c^	IC_50_ (μM) ^a^	Inhibition Type ^b^	*K_i_* (μM) ^c^
Sargahydroquinoic acid	5.14 ± 0.07 ^h^	Mixed	2.21	>100	-	-
Sargachromenol	11.80 ± 3.35 ^f^	Non-competitive	5.85	42.41 ± 3.09 ^h^	Non-competitive	33.95
Sargaquinoic acid	14.15 ± 0.02 ^f^	Mixed	5.20	96.17 ± 3.48 ^g^	Mixed	79.68
Ursolic acid ^d^	6.09 ± 0.02 ^g^					
Acarbose ^e^				210.76 ± 4.52 ^f^		

^a^ The 50% inhibitory concentration (µM) was calculated from a log-dose inhibition curve and is expressed as the mean ± SEM of triplicate experiments; ^b^ Inhibition type was determined by interpretation of the Dixon plot; ^c^ The inhibition constant (*K_i_*) was determined by interpretation of the Dixon plot; ^d,e^ Positive controls used in respective assays; ^f–h^ Mean with different letters are significantly different with Duncan’s test at *p* < 0.05.

**Table 3:** Binding site residues and docking scores of isolated compounds in PTP1B obtained using AutoDock 4.2.

Compound	Binding Energy ^a^ (kcal/mol)	No. of H-Bond ^b^	H-Bond Interacting Residues ^c^	van der Waals Bond Interacting Residues ^c^
Compound **23** ^d^ (catalytic inhibitor)	−11.23	11	Arg24, Tyr46, Asp48, Ser216, Ala217, Arg221, Arg254, Gln262	Ser28, Val49, Lys116, Lys120, Cys215, Ile219, Gly220, Met258, Gly259
Compound **2** ^d^ (allosteric inhibitor)	−10.98	2	Asn193, Glu276	Ala189, Leu192, Phe196, Gly277, Lys279, Phe280, Ile281, Met282
Sargahydroquinoic acid ^e^	−5.09	3	Arg24, Asp48, Gln262	Ile219, Ser28, Met258, Phe52, Asp29, Arg254, Gly259
	−5.95	2	Asn193, Lys197	Ala189, Leu192, Phe196, Glu276, Lys279, Phe280
Sargachromenol	−8.84	1	Asn193	Ser187, Pro188, Ala189, Leu192, Phe196, Lys197, Arg199, Glu200, Glu276, Phe280
Sargaquinoic acid ^e^	−3.13	3	Asp48, Lys116, Ala217	Arg24, Gln262, Thr263, Glu115, Arg221, Lys120, Ser216, Tyr46, Val49
	−6.83	1	Asn193	Ser187, Pro188, Ala189, Leu192, Phe196, Leu272, Glu276, Gly277, Phe280, Ile281

^a^ Estimated binding-free energy of the ligand receptor complex; ^b^ Number of hydrogen bonds between compounds and the active site of PTP1B; ^c^ All amino acid residues located 5 Å from the original enzyme/compound complex in the AutoDock 4.2 program; ^d^ Compound **23** (3-({5-[(*N*-acetyl-3-{4-[(carboxycarbonyl)(2-carboxyphenyl)amino]-1-naphthyl}-l-alanyl)amino]pentyl}oxy)-2-naphthoic acid) and compound **2** (3-(3,5-dibromo-4-hydroxy-benzoyl)-2-ethyl-benzofuran-6-sulfonic acid (4-sulfamoyl-phenyl)-amide) were used as positive ligands; ^e^ Sargahydroquinoic acid and sargaquinoic acid showed both type of catalytic (upper) and allosteric (lower) inhibition.

**Table 4:** Binding site residues and docking scores of isolated compounds in α-glucosidase using AutoDock 4.2.

Compound	Binding Energy ^a^ (kcal/mol)	No. of H-Bond ^b^	H-Bond Interacting Residues ^c^	van der Waals Bond Interacting Residues ^c^
Acarbose ^d^ (catalytic inhibitor)	−8.6	17	Asp69, Gln82, His112, Tyr158, Arg213, Asp215, Ser240, Asp242, Glu277, His280, Asp307, Asp352, Arg442	Tyr72, Lys156, Phe178, Val216, Gln279, Phe303, Arg315, His351, Gln353, Glu411
BIP ^d^ (allosteric inhibitor)	−6.75	1	Glu296	Trp15, Lys16, Asn259, Arg263, Val266, Gly269, Glu271, Ile272, Thr290, Ser291, Ala292,His295, Leu297, Ser298, Asp341, Cys342, Trp343
Sargahydroquinoic acid	−8.0	5	Glu296, Asn259, Thr274, His295	Trp15, Ile262, Arg270, Ile272, Val266, Ala292, Met273, Leu297, Ser298, Gly269, Glu271,Arg263
Sargachromenol	−7.3	1	Lys16	Lys13, Trp15, Asn259, Ile262, Arg263, Val266, Gly269, Arg270, Glu271, Ile272,Thr290, Ala292, Leu297, Ser298,Asp341
Sargaquinoic acid ^e^	−5.38	1	Asn350	Tyr158, Phe159, Phe178, Glu277, Gln279, His280, Phe303, Asp307, Phe314, Arg315, Asp352, Gln353, Glu411, Arg442
	−7.1	2	Ser291, Glu296	Asn259, Ile262,Arg263, Val266, Gly269, Arg270, Glu271,Ile272, Thr274, Leu297, Ala292, His295, Ser298

^a^ Estimated binding-free energy of the ligand receptor complex; ^b^ Number of hydrogen bonds between compounds and the active site of α-glucosidase; ^c^ All amino acid residues located 5 Å from the original enzyme/compound complex in the AutoDock 4.2 program; ^d^ Acarbose and BIP ((Z)-3-butylidenephthalide) were used as positive ligands; ^e^ Sargaquinoic acid showed both type of catalytic (upper) and allosteric (lower) inhibition.
